# Informal care in different European care systems: Effects of caregiving on mental health over time

**DOI:** 10.1371/journal.pone.0332498

**Published:** 2025-10-15

**Authors:** Leonie Börner, Ingo W. K. Kolodziej, Jürgen Wasem, Carina Abels

**Affiliations:** 1 Institute for Healthcare Management and Research, University of Duisburg Essen, Essen, Germany; 2 RWI – Leibniz Institute for Economic Research, Essen, Germany; 3 Fresenius University of Applied Sciences, Idstein, Germany; Universita degli Studi del Piemonte Orientale Amedeo Avogadro, ITALY

## Abstract

**Background:**

The study explores the impact of informal caregiving on mental health within different European care systems, recognizing the significant role of informal care due to demographic changes and the shortage of formal care options. The growing necessity for informal care is opposed to labor market demands and geographic mobility. A distinction is made between the “family effect” and the “care effect” on mental health, emphasizing the need to explore these impacts across different care systems longitudinally.

**Methods:**

Utilizing data from the Survey of Health, Aging, and Retirement in Europe (SHARE) across six waves, this study includes respondents aged 30 and older who participated in at least three waves. Participating countries were classified according to support services – outpatient care, payments for nursing care, obligation to support relatives – into the care systems implicit familism, explicit familism and optional familism. We employ least squares dummy variable (LSDV) regression followed by two-stage least squares (TSLS) regression to investigate intra-individual changes and the relationship between informal caregiving and mental health.

**Results:**

The sample comprises 5,761 individuals, with 2,800 individuals involved in informal caregiving across three defined care systems. LSDV-results show that caregiving significantly affects mental health in explicit familism for both genders and in implicit familism for women, increasing depressive symptoms as measured by the EURO-D score. These findings are not confirmed by TSLS-results. Instead TSLS-results show positive significant influence of informal care on mental health for both genders in implicit familism which include a reduction of EURO-D score and no significant results in explicit familism.

**Conclusion:**

The study highlights the differential impacts of informal caregiving on mental health across European care systems. The policy frameworks in implicit familism appear to benefit informal caregivers. Future research should further explore the dynamics of care systems and the role of policy interventions in supporting caregivers’ mental health.

## Background

Informal care is becoming increasingly important due to demographic change and oftentimes lack of formal care alternatives [1,2]. Informal care activities, which include any help with everyday tasks to family members, friends or people in the social network, should not only be seen as a supplement to outpatient and inpatient care within Europe, but as a cornerstone of long-term care in its own right [3]. Although the growing demand for informal care competes with developments in the labor market and geographical mobility, more than one sixth (17.3%) of the European population regularly provides informal care [2,4]. Thus, on the one hand, the group of informal caregivers relieves the burden on the care system through their caregiving activities. On the other hand, associated burden can also negatively impact health of informal caregivers, potentially increasing use of health care services in the long term. Based on the care stress model [5], previous studies identified factors with negative impact on mental health such as having a parallel employment relationship, financial worries, the relationship with the person being cared for and the intensity of care [2,3,6–8].

However, it is also important to differentiate between the “family effect” and the “care effect”, as they can influence mental health independently [9]. While the family effect describes the effects of caring *about* someone in need of care, the care effect comprises the effects of performing (informal) care activities (“caring *for* someone”) regardless of the relationship with the person in need of care.

In addition, the majority of longitudinal studies examined are limited to a specific country [10–12] or look at several European countries simultaneously without categorizing them regarding certain characteristics of the care systems [13,14]. Based on the care stress model, the effects on informal caregivers can vary depending on the support provided by the state and formal care services [5].

Accordingly, this study aims to examine the effects of informal care on mental health throughout Europe depending on the prevailing care systems. A unique contribution to previous literature is the heterogenous analysis by care systems while accounting for endogeneity: considering the respective care systems, we differentiate whether the various state support services have an influence on the development of stress and subsequently mental health issues in the context of informal care. From this, it can then be deduced which framework conditions favor or weaken the influence of informal care on mental health.

## Methods

### Data basis

We use data from the Survey of Health, Aging and Retirement in Europe (SHARE) waves 1 (2004/2005), 2 (2006/2007), 4 (2011/2012), 5 (2013), 6 (2015/2016) and 8 (2019/2020) (release 8.0.0) [15–21]. Waves 3 (2008/2009) and 7 (2017–2019) were excluded due to the separate design (retrospective survey of life history) and deviations in content. The data from the Corona Surveys were also omitted, as the pandemic prevailing at the time of the survey represents an independent influencing factor on mental health and thus limits comparability with the previous waves [22]. SHARE is a multidisciplinary panel study that surveys people aged 50 and over and their household members regarding health, socioeconomic status and social and family networks. The age limit of 50 years and older does not apply to the survey of household members. Therefore, household members surveyed in SHARE can also be younger than 50. To investigate longitudinal effects, countries that participated in all waves listed are included in the analysis which encompasses Austria, Belgium, Denmark, France, Germany, Italy, Spain, Sweden and Switzerland.

### Sample

The sample includes all respondents who participated in at least three consecutive waves within the six SHARE waves under review (1, 2, 4, 5, 6, 8). Although only people over the age of 50 are interviewed in the panel, there are also some observations of younger people. These come from the additional questioning of household members or relatives. People who participated in fewer than three waves or skipped one or more waves were excluded. Informal caregivers were defined as those who regularly provide help and support to other people outside and/or within their own household. Within the SHARE data, informal caregiving activities inside and outside the household include supportive activities, such as help with dressing, bathing, showering, etc., and refer to the last 12 months. Informal care activities within the household refer to daily or almost daily support services that were provided for at least three months. In contrast, the extent of informal care activities outside the household is surveyed separately and differentiated according to daily or almost daily, weekly, monthly, and less frequently. In line with Heger’s studies, only people who had at least one living parent at the time of the first survey were included [13]. This limitation offers the advantage of being able to differentiate specifically between the family and care effect. After excluding missing data 22,703 observations from 5,761 individuals are included.

### Study design

We make use of the longitudinal design of SHARE with a focus on intra-individual changes. We classify the countries according to the support services within the care system based on the explanations of Leitner and Haberkern and Szydlik [23,24].

Leitner categorises the countries into the types of implicit, explicit or optional familism depending on the state support services within care, while Haberkern and Szydlik differentiate between family-based and service-based care systems. The combination of the two categorisations offers the advantage that, in addition to the characteristic of how comprehensive the outpatient care provision is, two further characteristics can be considered, thus providing a broader spectrum for explanations. Leitner considers the characteristic of financial support for informal carers and Haberkern and Szydlik consider the (legal) obligation to support relatives. With regard to outpatient care provision, the characteristics of optional familism coincide with the service-based care system and those of implicit and explicit familism with the family-based care system (Table 1).

**Table 1:** Classification of care systems depending on outpatient care provision, payments for care for the elderly and the obligation to support relatives.

		Outpatient care	
		Comprehensive offer	Low offer
**Payments for nursing care (Leitner)**	Payments	optional familism	explicit familism
	No payments		implicit familism
**Obligation to support relatives (Haberkern and Szydlik)**	Obligation		family-based
	No Obligation	service-based	

The *family-based care system* is characterized by low coverage of outpatient care and a legal commitment for support from relatives. If payment for elderly care is also considered, a further distinction can be made between *implicit* and *explicit* familism. **Implicit familism** refers to the unspoken but deeply rooted expectations of family obligations that are not defined by formal rules or explicit agreements. These norms are based on the tacit assumption that family members support and care for each other. Such expectations are often culturally and socially embedded and are often taken for granted without extensive communication. There are little to no supportive measures from the state – neither payments for care for the elderly nor sufficient cover for outpatient care (Countries: Italy, Spain).

**Explicit familism** refers to a system within family relationships in which expectations and obligations are communicated clearly and directly. These obligations are often structured in the form of formal or informal agreements that specify the responsibilities of family members. All those involved are aware of their respective expectations and obligations. In contrast to implicit familism, however, the costs of care for the elderly are covered by the state, but the low level of cover provided by outpatient care services means that a large proportion of care work is still carried out by family members (Countries: Belgium, Germany, France, Austria).

In contrast, **optional familism** or **service based care-systems** describes a concept in which family support and responsibility are understood as voluntary decisions. In this model, there are no fixed or binding expectations regarding the provision of help within the family; rather, family members have the freedom to decide for themselves to what extent they want to provide or receive support. This form of family interaction is particularly common in modern, individualistic societies, where personal autonomy and individual freedom of choice are highly valued. A potential characteristic of this form is that it can lead to weaker family ties, as support is not taken for granted. The Scandinavian countries of Denmark and Sweden as well as Switzerland have these characteristics [8,23,24].

### Measures

Within the SHARE data the *EURO Depression Scale* (EURO-D) is used to survey mental health. EURO-D is based on five different instruments for diagnosing depression and records various symptoms of depression in a bundled form [25–29]. The scale consists of 12 items: depression, pessimism, suicidality, guilt, sleep, interest, irritability, appetite, tiredness, concentration, happiness and tearfulness. In summary, the items form a total score that ranges between 0 and 12. The higher the score, the more depressive symptoms are present. The minimum score of 0 is to be interpreted as “not depressed” and the maximum score of 12 as “very depressed” [30,31]. A score of 4 represents the cut-off and is used to classify the patient as suffering from a depressive disorder that requires therapeutic treatment [31–33]. EURO-D has internal consistency values between Cronbach’s alpha 0.58 and 0.80 [31,34]. Compliance with the quality criteria by the scale has been shown [32,33,35].

### Statistical analysis

We investigate intra-individual changes within the SHARE data in a linear panel data model described by


$$\begin{array}{c} {MH}_{it}=\text{ß}1{IC}_{it}+{\text{ß}2PH}_{it}+\text{ß}3X_{it}+u_{it}+c_{i} \end{array}$$ (1)

where ${MH}_{it}$ is the level of the dependent variable *mental health* for individual i in time t. ${IC}_{it}$ represents the independent variable, which records the individual’s i *informal care activities* in t. We closely follow Heger [13] by considering *parental health* ${PH}_{it}$ in the model to differentiate between family and care effects. Additional *control variables*, such as physical health, whether the mother and/or father are still alive as well as socio-demographic characteristics (age, marital status, household size, employment status, financial burdens) are represented by $X_{it}$. ß describes the associated regression coefficients. The error term $u_{it}$ includes all unobserved variables that change within individuals over time and is considered an *idiosyncratic error* in this context. The error term $c_{i}$, on the other hand, includes all variables that do not change within individuals, such as fundamental values, and is therefore referred to as the person effect. For a comprehensive overview of the variables, see S1 Table.

To estimate the panel data model in (1), a fixed-effects model (FEM) is first used and calculated using least squares dummy variable (LSDV) regression. The FEM assumes that the person effect $c_{i}$ is constant over time and that the explanatory variables are strictly exogenous in relation to the person effect $c_{i}$. As the sample includes individuals aged 30 and over, it can be assumed that the unobserved individual characteristics (e.g., altruism) are fixed and constant over time [36]. At the same time, the model allows for correlation between the explanatory variables and the time-invariant individual effect $c_{i}$ [37]. In the analyses, the different care systems as well as women and men are examined separately. The following therefore applies:


$${E(IC^{\prime }}_{i}c_{i})\neq \text{0},{E(PH^{\prime }}_{i}c_{i})\neq \text{0 and}{E(X^{\prime }}_{i}c_{i})\neq \text{0}.$$

In addition to the unobserved time-constant heterogeneity, time-variant endogeneity can pose a problem. For example, a significant adverse life event, especially like the death of a close relative – apart from the death of a parent – or a death in the circle of friends as well as witnessing this person at the end of life can both influence mental health and have an impact on the uptake of informal care for a parent [13]. Additional sources of endogeneity, stemming from simultaneous causality can lead to biased results. Consequently, the FEM is supplemented by an instrument variable estimation (IV) and analyzed using the two-stage least squares (TSLS) regression method. Following Heger’s study, the indicator that only one parent is alive (single parent) is used as an instrument for informal caregiving in order to address the endogeneity problem [13]. As a single parent can no longer fall back on the support of a spouse in the event of needing care, this increases the probability that informal care will be provided by the child [13,38]. Furthermore, the need for informal care no longer applies if neither parent is alive. For the instrument of a single parent (SP) to be valid, it must not influence the child’s mental health directly, but only through the provision of informal care. Secondly, the instrument must not show any correlation with the error term $u_{it}$. The following therefore applies:


$${corr(CG}_{it},{SP}_{it})\neq 0,E\left(u_{it}|{SP}_{it}\right)=0$$

Having a single parent has no additional direct effect on the mental health of informal caregiving children, as both parental health and the loss of a parent are already considered. We formally test the validity of the instrument using an F-test in the first stage of the TSLS regression. If the associated F statistic is greater than ten, a valid instrument can be assumed [39].

## Results

### Study population

The sample comprises 3,377 women and 2,384 men (n = 5,761), including 2,800 individuals who have provided informal care at least once. Of these, 19.04% of informal caregivers can be classified as implicit familism, 48.57% as explicit familism and 32.39% as optional familism (Table 2). Informal caregivers in the implicit familism show the highest EURO-D score and the highest proportions for classification in a depressive disorder. The values for mental health are lowest in optional familism. Implicit familism shows the highest percentage values for the case that the mother or father is in poor health compared to the other care systems. The lowest number of chronic illnesses of informal caregivers is recorded in the optional familism. A lower proportion of informal caregivers in the optional familism are married or in a partnership and the household size is smaller than that of people in the other two care systems. Optional familism has the highest proportion of employed persons and the lowest proportion of financial burdens (Table 2).

**Table 2:** Characteristics of women (W) and men (M) providing informal care by care system.

	All Informal caregivers		Implicit familism		Explicit familism		Optional familism	
	W	M	W	M	W	M	W	M
*Mental health*								
EURO-D score 4 + depressive symptoms	2.46 0.28	1.59 0.13	2.80 0.33	1.77 0.18	2.56 0.30	1.70 0.15	2.09 0.21	1.36 0.09
*Parental information*								
Single parent Mother in fair or poor health Father in fair or poor health Mother alive Father alive	0.76 0.40 0.15 0.91 0.33	0.78 0.38 0.14 0.89 0.33	0.78 0.47 0.16 0.90 0.32	0.79 0.49 0.20 0.87 0.34	0.76 0.40 0.16 0.91 0.33	0.76 0.36 0.13 0.90 0.34	0.75 0.35 0.15 0.90 0.35	0.80 0.34 0.13 0.88 0.31
*Health*								
Number of chronic diseases ADL limitations IADL limitations	1.13 0.04 0.07	1.10 0.02 0.02	1.19 0.04 0.06	1.07 0.01 0.02	1.19 0.04 0.07	1.22 0.04 0.02	0.99 0.04 0.07	0.95 0.01 0.02
*Sociodemographics*								
Age Married Household size Partner in household Employed Financial difficulties	56.40 0.77 2.39 0.80 0.59 0.27	57.08 0.81 2.52 0.84 0.69 0.23	56.24 0.87 2.96 0.86 0.41 0.51	57.69 0.84 3.03 0.86 0.63 0.49	56.38 0.76 2.32 0.79 0.56 0.25	56.61 0.82 2.49 0.84 0.62 0.23	56.53 0.73 2.14 0.78 0.75 0.12	57.45 0.79 2.32 0.83 0.81 0.10
Observations	1834	966	373	160	891	469	570	337

### LSDV regression

Table 3 shows the results of the LSDV regression. In **implicit familism**, informal caregiving causes an increase in the EURO-D score of 0.37 points and a 5% increase in the probability of suffering from four or more depressive symptoms and the mother’s poor state of health leads to an increase in the EURO-D score of 0.23 points for women while 5% increase in the probability of suffering from four or more depressive symptoms is shown for both genders. Physical health of the caregiver negatively affects mental health of both genders, ranging from 0.20 to 0.80 points and 4–14% for women and 0.19 to 0.84 points as well as 2–13% for men. In addition, the probability of experiencing four or more depressive symptoms falls by 18% if women and 24% if men are married or living a partnership. This factor also leads to a reduction of the EURO-D score by 1.94 points for men. A larger household size increase the EURO-D score by 0.14 points for women.

**Table 3:** LSDV-results of the effect of informal caregiving on women’s and men’s mental health.

	Women						Men					
	EURO-D score			4 + depressive symptoms			EURO-D score			4 + depressive symptoms		
	Implicit familism	Explicit familism	Optional familism	Implicit familism	Explicit familism	Optional familism	Implicit familism	Explicit familism	Optional familism	Implicit familism	Explicit familism	Optional familism
Informal care	0.366***	0.109*	0.091	0.050**	0.016	0.012	0.049	0.169**	−0.064	0.022	0.003	−0.012
	(0.124)	(0.060)	(0.065)	(0.025)	(0.014)	(0.016)	(0.139)	(0.073)	(0.077)	(0.028)	(0.016)	(0.017)
Mother in fair or poor health	0.225*	0.278***	−0.048	0.052*	0.053***	−0.007	0.186	0.107	0.199**	0.049**	0.023	0.015
	(0.131)	(0.065)	(0.076)	(0.027)	(0.016)	(0.018)	(0.119)	(0.072)	(0.082)	(0.024)	(0.016)	(0.018)
Father in fair or poor health	0.135	0.314***	0.158	0.014	0.028	0.022	0.093	0.040	0.058	0.014	−0.028	0.014
	(0.224)	(0.106)	(0.118)	(0.046)	(0.025)	(0.029)	(0.196)	(0.114)	(0.128)	(0.039)	(0.025)	(0.028)
Mother alive	−0.032	−0.377***	−0.082	−0.029	−0.071***	−0.002	−0.146	−0.073	−0.240**	−0.021	0.001	−0.012
	(0.172)	(0.090)	(0.098)	(0.035)	(0.021)	(0.024)	(0.150)	(0.094)	(0.104)	(0.030)	(0.021)	(0.023)
Father alive	−0.332	−0.385***	−0.191	−0.067	−0.052*	−0.061**	0.002	0.106	0.122	−0.030	0.041	0.034
	(0.213)	(0.116)	(0.125)	(0.044)	(0.028)	(0.031)	(0.186)	(0.121)	(0.135)	(0.037)	(0.027)	(0.029)
Number of chronic diseases	0.204***	0.144***	0.128***	0.035***	0.029***	0.021***	0.190***	0.124***	0.098***	0.022**	0.009	0.008
	(0.047)	(0.025)	(0.031)	(0.010)	(0.006)	(0.008)	(0.044)	(0.027)	(0.033)	(0.009)	(0.006)	(0.007)
ADL limitations	0.700***	0.424***	0.321**	0.080*	0.089***	0.034	0.429*	0.420***	0.516***	0.101**	0.076***	0.056*
	(0.231)	(0.111)	(0.134)	(0.047)	(0.026)	(0.033)	(0.227)	(0.111)	(0.151)	(0.045)	(0.025)	(0.033)
IADL limitations	0.797***	0.416***	0.580***	0.147***	0.075***	0.076***	0.838***	0.595***	0.477***	0.125***	0.094***	0.106***
	(0.176)	(0.089)	(0.108)	(0.036)	(0.021)	(0.026)	(0.222)	(0.112)	(0.146)	(0.044)	(0.025)	(0.032)
Age	−0.023	−0.036	−0.214***	−0.012	−0.004	−0.040***	−0.034	−0.080	−0.248***	0.001	−0.013	−0.028*
	(0.090)	(0.051)	(0.055)	(0.018)	(0.012)	(0.013)	(0.098)	(0.061)	(0.067)	(0.019)	(0.013)	(0.015)
Married	−0.634	−0.630***	−0.205	−0.182*	−0.083*	−0.013	−1.935***	0.088	−0.125	−0.237***	−0.034	0.028
	(0.474)	(0.193)	(0.198)	(0.097)	(0.046)	(0.048)	(0.455)	(0.204)	(0.221)	(0.090)	(0.045)	(0.048)
Household size	0.135*	−0.004	−0.084	0.022	0.007	−0.020	−0.038	−0.042	−0.092	−0.007	0.005	−0.021
	(0.077)	(0.054)	(0.059)	(0.016)	(0.013)	(0.014)	(0.070)	(0.051)	(0.060)	(0.014)	(0.011)	(0.013)
Partner in household	−0.304	0.017	−0.244	0.083	−0.024	−0.035	0.349	−0.104	−0.209	0.041	−0.002	−0.041
	(0.415)	(0.166)	(0.184)	(0.085)	(0.040)	(0.045)	(0.346)	(0.171)	(0.207)	(0.069)	(0.038)	(0.045)
Employed	0.052	0.086	0.187**	0.050	0.016	0.051***	0.163	0.044	0.026	0.004	0.036**	−0.015
	(0.168)	(0.072)	(0.077)	(0.034)	(0.017)	(0.019)	(0.131)	(0.078)	(0.083)	(0.026)	(0.017)	(0.018)
Financial worries	0.172	0.196***	0.101	0.036	0.053***	0.008	0.139	0.182**	0.062	0.028	0.047***	0.035
	(0.112)	(0.068)	(0.098)	(0.023)	(0.016)	(0.024)	(0.101)	(0.079)	(0.113)	(0.020)	(0.017)	(0.025)
Observations	2772	6508	4043	2772	6508	4043	1992	4515	2873	1992	4515	2873
R-squared	0.022	0.011	0.015	0.018	0.009	0.009	0.027	0.014	0.016	0.018	0.009	0.010
Unique individuals	693	1691	993	693	1691	993	504	1173	707	504	1173	707

Robust standard errors are in parentheses.

*** *p* < 0.01; ** *p* < 0.05; * *p* < 0.1.

In **explicit familism**, informal caregiving leads to a significant increase in the EURO-D score of 0.17 points for men and 0.11 points for women. For women, an impaired state of parental health leads to an increase of the EURO-D score by 0.28 to 0.31 points and poor maternal health increases probability of depressive illness by 5%. The presence of a parent reduces the EURO-D score by around 0.38 points and the probability of having four or more depressive symptoms by 5–7%. The parameters of one’s own physical health influence mental health of women and men, with restrictions in ADL and IADL leading to slightly greater increases in the measurement parameters for mental health (0.42 to 0.60 points and 8–9%) than the number of chronic illnesses (0.13 to 0.14 points and 3%). Being married or living in a partnership lowers the EURO-D score by 0.63 points and the probability of suffering from four or more depressive symptoms by 8% for women. Being self-employed or employed increases this probability by around 4% for men. The presence of financial worries, on the other hand, leads to an increase in both parameters for women and men (0.18 to 0.20 points and 5% respectively).

In **optional familism**, the presence of the father lowers the classification of depressive illness by 6% for women, while the employment status (self-employed or employed) causes an increase (0.19 points and 5%). For men, a poor state of maternal health leads to an increase in the EURO-D score by 0.20 points and the presence of the mother leads to a decrease in the EURO-D score by 0.24 points. Impairments in caregiver physical health, increase the EURO-D score by 0.10 to 0.58 points and the probability of suffering from four or more depressive symptoms by 2–11%. Increasing age causes a decrease in the mental health measurement parameters (0.21 points and 4% for women and 0.25 points and 3% for men).

### TSLS regression

To counter possible distortions of the results within the LSDV regression due to time-varying endogeneity, we use the presence of a single living parent as an instrument for informal caregiving. For each care system, we test the strength of the instrument (Table 4). The results from the first stage are highly significant and show that the probability of providing informal care in implicit familism increases by 23.0% for women and 13.4% for men when switching from two to one living parent. In explicit familism, the instrument increases the probability of providing informal care by 27.3% for women and 19.7% for men. Even higher values are recorded in optional familism with an increase of 29.4% for women and 23.8% for men. The F-statistic is above ten for all care systems and both genders.

**Table 4:** Results of the F-test to test the quality of the instrument variable by care system.

	Implicit familism	Explicit familism	Optional familism
*Women*			
Single Parent	0.230*** (0.013)	0.273*** (0.008)	0.294*** (0.011)
Observations	2772	6508	4043
R-squared	0.063	0.089	0.092
Unique indiviuals	693	1691	993
F-statistic	17.65	33.98	26.05
*Men*			
Single Parent	0.134*** (0.012)	0.197*** (0.008)	0.238*** (0.012)
Observations	1992	4515	2873
R-squared	0.037	0.064	0.076
Unique indiviuals	504	1173	707
F-statistic	11.56	23.26	19.90

Robust standard errors are in parentheses.

*** *p* < 0.01; ** *p* < 0.05; * *p* < 0.1.

The results of the second stage in TSLS regression only show a marginally significant influence of informal care on mental health for women and men in implicit familism (Table 5). For women, the probability of suffering from four or more depressive symptoms is reduced by 18% if informal caregiving activities are carried out. For men, providing informal care lowers the EURO-D score by 1.37 points and the probability of suffering from four or more depressive symptoms by 29%. In explicit familism, informal caregiving has no influence on women’s mental health. In line with the LSDV regression, mental health of women in implicit familism is influenced by the mother’s impaired state of health, their own physical health, marital status and household size. In explicit familism, both the mother’s and the father’s poor state of health have an influence on mental health of women. We also find significant results for the presence of a parent, one’s own physical health, marital status and financial burdens. The optional familism similarly shows comparable results to the LSDV regression. The presence of a father, own physical health, age and employment status influence women’s mental health and own physical health has an influence on mental health of men across all care systems. In implicit familism, marital status also leads to a change in mental health. Employment status and the presence of financial burdens only have an influence on mental health in explicit familism, while an impaired state of health of the mother influences the mental health of men in both implicit and optional familism. Furthermore, an influence of age on mental health can only be demonstrated in the optional familism.

**Table 5:** TSLS-results of the effect of informal caregiving on women’s and men’s mental health.

	Women							Men						
		EURO-D score			4 + depressive symptoms				EURO-D score			4 + depressive symptoms		
		Implicit familism	Explicit familism	Optional familism	Implicit familism	Explicit familism	Optional familism		Implicit familism	Explicit familism	Optional familism	Implicit familism	Explicit familism	Optional familism
Informal care		−0.498	0.085	−0.219	−0.175*	−0.034	−0.008		−1.366*	0.230	−0.327	−0.287*	0.064	−0.023
		(0.479)	(0.201)	(0.211)	(0.098)	(0.048)	(0.052)		(0.743)	(0.302)	(0.284)	(0.147)	(0.067)	(0.062)
Mother in fair or poor health		0.271**	0.286***	−0.042	0.059**	0.054***	−0.006		0.188	0.108	0.196**	0.051**	0.023	0.015
		(0.131)	(0.065)	(0.075)	(0.027)	(0.016)	(0.018)		(0.118)	(0.072)	(0.081)	(0.023)	(0.016)	(0.018)
Father in fair or poor health		0.162	0.329***	0.150	0.016	0.030	0.021		0.055	0.044	0.051	0.006	−0.028	0.013
		(0.224)	(0.106)	(0.118)	(0.046)	(0.025)	(0.029)		(0.197)	(0.114)	(0.128)	(0.039)	(0.025)	(0.028)
Mother alive		0.094	−0.370***	−0.020	0.002	−0.061***	0.002		−0.038	−0.072	−0.200*	0.003	−0.006	−0.011
		(0.183)	(0.096)	(0.104)	(0.037)	(0.023)	(0.026)		(0.161)	(0.100)	(0.113)	(0.032)	(0.022)	(0.025)
Father alive		−0.329	−0.386***	−0.185	−0.067	−0.054*	−0.060**		0.039	0.109	0.119	−0.021	0.042	0.033
		(0.214)	(0.116)	(0.125)	(0.044)	(0.028)	(0.031)		(0.187)	(0.121)	(0.135)	(0.037)	(0.027)	(0.029)
Number of chronic diseases		0.208***	0.144***	0.130***	0.036***	0.029***	0.021***		0.189***	0.125***	0.096***	0.022**	0.009	0.008
		(0.047)	(0.025)	(0.031)	(0.010)	(0.006)	(0.008)		(0.044)	(0.027)	(0.033)	(0.009)	(0.006)	(0.007)
ADL limitations		0.698***	0.426***	0.317**	0.079*	0.089***	0.034		0.430*	0.418***	0.518***	0.102**	0.076***	0.057*
		(0.231)	(0.111)	(0.134)	(0.047)	(0.026)	(0.033)		(0.227)	(0.111)	(0.151)	(0.045)	(0.025)	(0.033)
IADL limitations		0.781***	0.415***	0.580***	0.143***	0.075***	0.076***		0.831***	0.591***	0.482***	0.123***	0.094***	0.106***
		(0.177)	(0.089)	(0.108)	(0.036)	(0.021)	(0.026)		(0.222)	(0.112)	(0.146)	(0.044)	(0.025)	(0.032)
Age		0.015	−0.033	−0.200***	−0.003	−0.001	−0.039***		0.011	−0.083	−0.235***	0.011	−0.015	−0.028*
		(0.092)	(0.052)	(0.055)	(0.019)	(0.012)	(0.014)		(0.100)	(0.062)	(0.068)	(0.020)	(0.014)	(0.015)
Married		−0.646	−0.623***	−0.205	−0.185*	−0.081*	−0.013		−1.943***	0.083	−0.128	−0.238***	−0.034	0.028
		(0.475)	(0.193)	(0.198)	(0.097)	(0.046)	(0.048)		(0.454)	(0.204)	(0.221)	(0.090)	(0.045)	(0.048)
Household size		0.145*	−0.003	−0.086	0.024	0.008	−0.020		−0.038	−0.035	−0.094	−0.007	0.005	−0.021
		(0.077)	(0.054)	(0.198)	(0.016)	(0.013)	(0.014)		(0.070)	(0.051)	(0.060)	(0.014)	(0.011)	(0.013)
Partner in household		−0.334	0.014	−0.247	0.079	−0.025	−0.035		0.363	−0.113	−0.197	0.044	−0.001	−0.040
		(0.416)	(0.167)	(0.184)	(0.085)	(0.040)	(0.045)		(0.346)	(0.171)	(0.207)	(0.069)	(0.038)	(0.045)
Employed		0.039	0.086	0.190**	0.049	0.017	0.052***		0.158	0.041	0.032	0.004	0.035**	−0.014
		(0.169)	(0.072)	(0.077)	(0.034)	(0.017)	(0.019)		(0.131)	(0.078)	(0.083)	(0.026)	(0.017)	(0.018)
Financial worries		0.180	0.196***	0.100	0.037	0.052***	0.007		0.129	0.185**	0.059	0.026	0.048***	0.035
		(0.113)	(0.068)	(0.098)	(0.023)	(0.016)	(0.024)		(0.101)	(0.079)	(0.113)	(0.020)	(0.017)	(0.025)
Observations		2772	6508	4043	2772	6508	4043		1992	4515	2873	1992	4515	2873
R-squared		0.020	0.011	0.015	0.018	0.009	0.009		0.028	0.013	0.016	0.019	0.009	0.010
Unique individuals		693	1691	993	693	1691	993		504	1173	707	504	1173	707

Robust standard errors are in parentheses.

*** *p* < 0.01; ** *p* < 0.05; * *p* < 0.1.

## Discussion

In this study, we investigate the influence of informal caregiving on mental health within different care systems. The results of the LSDV regression show a negative influence of informal caregiving on mental health for women in implicit and explicit familism and for men only in explicit familism. Within the subsequent TSLS regression, on the other hand, the results show a positive influence of informal caregiving on mental health for men and women in implicit familism, while no influence was detected in explicit and optional familism.

We interpret these results based on the different characteristics of the care systems: the non-significant results for optional familism reflect ample state support in this system. Taking up informal care activities in these cases is almost voluntary and presumably more likely to be practiced by people who feel healthy and up to the task themselves. The associated descriptive results of informal caregivers in the various care systems support this consideration. Informal caregivers in optional familism are in comparatively better mental and physical health. This finding is consistent with the studies by Estrada Fernández and colleagues: they found that informal caregivers in Nordic countries, which are predominantly characterized by optional familism, are less depressed and happier. In contrast, informal caregivers from Mediterranean countries such as Spain and France, which are characterized by the family-based care system, showed impaired health [40].

As there is little coverage of outpatient care and an obligation for support from relatives in both implicit and explicit familism, the provision of informal care is not always voluntary. Consequently, informal care work can be seen as an additional burden. However, we could only observe this additional burden in the LSDV regression. Our results are in line with the findings of Kaschowitz and Brandt who show significant differences in health between informal caregivers and non-carers in family-based context using FEM. The informal caregivers showed a poorer state of health [8]. However, while FEM analysis can be informative, we use TSLS estimation to highlight the effects of informal care as a function of the care systems using this supplementary approach. The differences in results between FEM and TSLS analysis indicate remaining endogeneity bias in FEM. FEM results still capture unmeasured stressors as part of the caregiving effect, like financial strain or job loss, that vary over time and simultaneously worsen mental health and increase caregiving likelihood. Bias can also stem from reverse causality, i.e., if individuals with mental health problems become caregivers. TSLS disentangles the causal effect and eliminates this bias. The reversal of the sign highlights the necessity of examining different care systems with underlying mentality in these countries, as it depends on why someone becomes a caregiver: The effects of informal care on mental health are positive in implicit familism using TSLS regression. The TSLS results denote caregiving effects exogenously originating from the care needs of dependent single parents and imply that in a care system in which informal care is provided by relatives, informal caregiving could lead to fulfillment and satisfaction. Informal caregivers may feel useful and experience gratitude and appreciation from the person receiving care. However, results may further differ by caregiving intensity, as has been found, e.g., for the US, with more intensive caregiving leading to increased anxiety and worse relationship quality as well as less satisfaction that the care recipient is well-cared for [41]. Moreover, Heger (2017) does only find differences between countries in one fifth of the cases, when she groups countries by geographic region and according to spending on LTC. This difference to the results of our study might stem from different group definitions and the use of different waves of SHARE data. Together with the reversal of the sign of the coefficients between FEM and TSLS, it can also reflect a change over time in norms, with caregiving being more of a choice nowadays, including in countries with implicit familism.

Our analysis has various limitations. The study was restricted to people aged 30 and over. Statistics show that, on average, around 11% of 15–29-year-olds in Europe provide informal care [42]. Informal care can also be a risk factor for young adults, who are in an age period characterized by many changes and developments, which can have consequences for their mental health [43,44]. For example, Fleitas Alfonzo and colleagues showed in their review that young informal caregivers participate in fewer social interactions, isolate themselves and consequently have poorer mental health than their peer group [45].

Furthermore, we restrict our sample to individuals providing care to a parent. Providing informal care to a partner, friend or other person may have a different impact on mental health, as the circumstances and reasons for providing informal care may also differ. For example, people who have a spouse or partner in need of care are more likely to feel obliged to provide informal care, are more likely to take on more intensive care activities and consequently have greater mental health impairments [9,46].

In addition, while the TSLS estimation shows highly relevant and significant first stage, future research could take advantage of larger sample sizes: due to the differentiated consideration of the respective care systems, comparatively small samples are available and differing sample sizes of the care systems hinder comparability of results between the care systems.

The prevailing care systems can also be a combination or a hybrid and not always be clearly assigned to one of the three categories [47]. Accordingly, the countries were allocated to the care system categories based on the closest match of certain characteristics. On the other hand, a one-off allocation was made based on the current care system characteristics and possible changes to the care system over time were not considered. Care reforms, however, may not only have an impact on the basic care system structures (formal care services, state support services), but can also have a secondary influence on informal caregivers and their (mental) state of health.

To identify the influence of informal care on mental health, various control variables based on Pearlin’s care stress model were considered in addition to the care activity. However, due to the predefined queries within the SHARE data and the resulting information on each person, not all aspects of the caregiving stress model could be included in the analysis.

Finally, the results from our TSLS estimations are local average treatment effects (LATE) and apply to those whose caregiving status is altered by the instrument.

## Conclusion

We do not find significant effects of informal care on number of depressive symptoms or probability of reporting four or more depressive symptoms in explicit and optional familism, while we can identify a positive influence on mental health in implicit familism, when accounting for time-varying endogeneity in TSLS regression. Accordingly, the framework conditions in implicit familism appear to benefit mental health of informal caregivers. While a distinctive contribution of this paper is the heterogenous analysis by care systems, future research could look in more detail at the various support services within a care system and examine which services are accepted by informal caregivers and support them in their situation. Overall, caregiver buffers within care systems play an important role in ameliorating mental health outcomes and help cope with caregiver stressors [5,41,48]. Decision-makers should take these results into account when formulating adaptations in the respective long-term care systems. The different support systems are an expression of the mentality and social norms in these countries which needs to be considered if care reforms are assessed in individual countries. The different mentalities are already taken into account, for example, when measuring quality of life using EQ-5D. Due to different social norms, a loss of independence, for example, is weighted differently depending on the country when calculating quality of life.

With regard to the implementation of reforms, the results of the study demonstrate that the structures of other countries with different care systems cannot be transferred without taking into account the social aspects of both countries.

## Supporting information

S1 Table Description of used variables. (PDF)

## Abbreviation

ADL: activities of daily living
β: regression coefficient
c: person effect
corr: correlation
EURO-D: EURO depression scale
FEM: fixed effects model
i: individual
IADL: instrumental activities of daily living
IC: informal care activities
IV: instrument variable estimation
LSDV: Least square dummy variable
MH: mental health
PH: parental health
SES: socioeconomic status
SHARE: Survey of Health, Aging and Retirement in Europe
SP: Single parent
SJR: Scientific Journal Ranking
t: time period
TSLS: Two Stages Least Squares
u: idiosyncratic error
X: control variables

## References

[1] Corselli-Nordblad L, Strandell H. Ageing Europe: looking at the lives of older people in the EU: 2020 edition. 20 ed. Luxembourg: Publications Office of the European Union. 2020.

[2] Carabott J, Kobal Straus K, Guštin E, editors. 2021 Long-term care report: Trends, challenges and opportunities in an ageing society: joint report prepared by the Social Protection Committe (SPC) and the European Commission (DG EMPL). Luxembourg: Publications Office of the European Union; 2021.

[3] OECD. Health at a glance 2021: OECD indicators. Paris: OECD Publishing. 2021.

[4] Eurostat. Persons providing informal care or assistance at least once a week by sex, age and degree of urbanisation [online data code: hlth_ehis_ic1u__custom_8024648]. 2022. [cited 23 Oct 2023]. https://ec.europa.eu/eurostat/databrowser/view/hlth_ehis_ic1u__custom_8024648/default/table?lang=en&page=time:2019

[5] Pearlin LI, Mullan JT, Semple SJ, Skaff MM. Caregiving and the stress process: an overview of concepts and their measures. Gerontologist. 1990;30(5):583–94. doi: 10.1093/geront/30.5.583 2276631

[6] Bom J, Bakx P, Schut F, van Doorslaer E. The Impact of Informal Caregiving for Older Adults on the Health of Various Types of Caregivers: A Systematic Review. Gerontologist. 2019;59(5):e629–42. doi: 10.1093/geront/gny137 30395200 PMC6850889

[7] Pinquart M, Sörensen S. Associations of stressors and uplifts of caregiving with caregiver burden and depressive mood: a meta-analysis. J Gerontol B Psychol Sci Soc Sci. 2003;58(2):P112-28. doi: 10.1093/geronb/58.2.p112 12646594

[8] Kaschowitz J, Brandt M. Health effects of informal caregiving across Europe: A longitudinal approach. Soc Sci Med. 2017;173:72–80. doi: 10.1016/j.socscimed.2016.11.036 27930918

[9] Bom J, Bakx P, Schut F, van Doorslaer E. Health effects of caring for and about parents and spouses. The Journal of the Economics of Ageing. 2019;14:100196. doi: 10.1016/j.jeoa.2019.100196

[10] Lacey RE, McMunn A, Webb E. Informal caregiving patterns and trajectories of psychological distress in the UK Household Longitudinal Study. Psychol Med. 2019;49(10):1652–60. doi: 10.1017/S0033291718002222 30205848 PMC6601356

[11] Stratmann M, Forsell Y, Möller J, Liang Y. Informal care and the impact on depression and anxiety among Swedish adults: a population-based cohort study. BMC Public Health. 2021;21(1):1263. doi: 10.1186/s12889-021-11246-1 34187429 PMC8243546

[12] Urwin S, Lau Y, Grande G, Sutton M. Informal caregiving, time use and experienced wellbeing. Health Economics. 2022;32(2):356–74. doi: 10.1002/hec.4624 36303421 PMC10092671

[13] Heger D. The Mental Health of Children Providing Care to their Elderly Parent. Health Econ. 2017;26(12):1617–29. doi: 10.1002/hec.3457 27917556

[14] Hiel L, Beenackers MA, Renders CM, Robroek SJW, Burdorf A, Croezen S. Providing personal informal care to older European adults: should we care about the caregivers’ health?. Prev Med. 2015;70:64–8. doi: 10.1016/j.ypmed.2014.10.028 25450490

[15] Bergmann M, Kneip T, Luca G de, Scherpenzeel A. Survey participation in the Survey of Health, Ageing and Retirement in Europe (SHARE), Wave 1-7. Based on Release 7.0.0. Munich: MEA, Max Planck Institute for Social Law and Social Policy: SHARE Working Paper Series 41-2019; 2019.

[16] Börsch-Supan A. Survey of Health, Ageing and Retirement in Europe (SHARE) Wave 1. Release version: 8.0.0. SHARE-ERIC; 2022.

[17] Börsch-Supan A. Survey of Health, Ageing and Retirement in Europe (SHARE) Wave 2. Release version: 8.0.0. SHARE-ERIC; 2022.

[18] Börsch-Supan A. Survey of Health, Ageing and Retirement in Europe (SHARE) Wave 4. Release version: 8.0.0. SHARE-ERIC; 2022.

[19] Börsch-Supan A. Survey of Health, Ageing and Retirement in Europe (SHARE) Wave 5. Release version: 8.0.0. SHARE-ERIC; 2022.

[20] Börsch-Supan A. Survey of Health, Ageing and Retirement in Europe (SHARE) Wave 6. Release version: 8.0.0. SHARE-ERIC; 2022.

[21] Börsch-Supan A. Survey of Health, Ageing and Retirement in Europe (SHARE) Wave 8. Release version: 8.0.0. SHARE-ERIC; 2022.

[22] OECD. A new benchmark for mental health systems: Tackling the social and economic costs of mental ill-health. Paris: OECD Publishing. 2021.

[23] Leitner S. Varieties of familialism: The caring function of the family in comparative perspective. European Societies. 2003;5(4):353–75. doi: 10.1080/1461669032000127642

[24] Haberkern K, Szydlik M. Pflege der Eltern – Ein europäischer Vergleich*. Koelner ZSoziol.uSozPsychol. 2008;60(1):82–105. doi: 10.1007/s11577-008-0004-y

[25] Copeland JR, Dewey ME, Griffiths-Jones HM. A computerized psychiatric diagnostic system and case nomenclature for elderly subjects: GMS and AGECAT. Psychol Med. 1986;16(1):89–99. doi: 10.1017/s0033291700057779 3515380

[26] Gurland B, Golden RR, Teresi JA, Challop J. The SHORT-CARE: an efficient instrument for the assessment of depression, dementia and disability. J Gerontol. 1984;39(2):166–9. doi: 10.1093/geronj/39.2.166 6699370

[27] Radloff LS. The CES-D Scale. Applied Psychological Measurement. 1977;1(3):385–401. doi: 10.1177/014662167700100306

[28] Zung WW. A self-rating depression scale. Arch Gen Psychiatry. 1965;12:63–70. doi: 10.1001/archpsyc.1965.01720310065008 14221692

[29] Asberg M, Perris C, Schalling D, Sedvall G. CRPS: Development and applications of a psychiatric rating scale. Acta Psychiatrica Scandinavica. 1978;271:1–69. 10.1111/j.1600-0447.1978.tb02357.x 277059

[30] Prince MJ, Beekman AT, Deeg DJ, Fuhrer R, Kivela SL, Lawlor BA, et al. Depression symptoms in late life assessed using the EURO-D scale. Effect of age, gender and marital status in 14 European centres. Br J Psychiatry. 1999;174:339–45. doi: 10.1192/bjp.174.4.339 10533553

[31] Prince MJ, Reischies F, Beekman AT, Fuhrer R, Jonker C, Kivela SL, et al. Development of the EURO-D scale--a European, Union initiative to compare symptoms of depression in 14 European centres. Br J Psychiatry. 1999;174:330–8. doi: 10.1192/bjp.174.4.330 10533552

[32] Castro-Costa E, Dewey M, Stewart R, Banerjee S, Huppert F, Mendonca-Lima C, et al. Prevalence of depressive symptoms and syndromes in later life in ten European countries: the SHARE study. Br J Psychiatry. 2007;191:393–401. doi: 10.1192/bjp.bp.107.036772 17978318

[33] Castro-Costa E, Dewey M, Stewart R, Banerjee S, Huppert F, Mendonca-Lima C, et al. Ascertaining late-life depressive symptoms in Europe: an evaluation of the survey version of the EURO-D scale in 10 nations. The SHARE project. Int J Methods Psychiatr Res. 2008;17(1):12–29. doi: 10.1002/mpr.236 18286461 PMC6878368

[34] Prince M. The development of the EURO-D scale. In: Copeland JRM, Abou-Saleh MT, Blazer DG, editors. Principles and Practice of Geriatric Psychiatry. London: John Wiley & Sons; 2002. p. 159–160. doi: 10.1002/0470846410.ch30

[35] Courtin E, Knapp M, Grundy E, Avendano‐Pabon M. Are different measures of depressive symptoms in old age comparable? An analysis of the CES‐D and Euro‐D scales in 13 countries. Int J Methods Psych Res. 2015;24(4):287–304. doi: 10.1002/mpr.1489 PMC6878602 26314384

[36] Terracciano A, McCrae RR, Costa PT Jr. Intra-individual change in personality stability and age. Journal of Research in Personality. 2010;44(1):31–7. doi: 10.1016/j.jrp.2009.09.006 20305728 PMC2839250

[37] Wooldridge JM. Econometric Analysis of Cross Section and Panel Data. Cambridge: MIT Press. 2002.

[38] Wolf DA, Raissian KM, Grundy E. Parental disability, parent care, and offspring mental health outcomes. Eur J Ageing. 2015;12(3):175–85. doi: 10.1007/s10433-015-0339-y 28804353 PMC5549236

[39] Staiger D, Stock JH. Instrumental Variables Regression with Weak Instruments. Econometrica. 1997;65(3):557. doi: 10.2307/2171753

[40] Estrada Fernández ME, Gil Lacruz AI, Gil Lacruz M, Viñas López A. Informal care. European situation and approximation of a reality. Health Policy. 2019;123(12):1163–72. doi: 10.1016/j.healthpol.2019.09.007 31606144

[41] Kolodziej IWK, Coe NB, Van Houtven CH. The Impact of Care Intensity and Work on the Mental Health of Family Caregivers: Losses and Gains. J Gerontol B Psychol Sci Soc Sci. 2022;77(Suppl_1):S98–111. doi: 10.1093/geronb/gbac031 35191980 PMC9122646

[42] Eurostat. Persons providing informal care or assistance at least once a week by sex, age and degree of urbanisation [online data code: hlth_ehis_ic1u__custom_8024987]. 2022. [cited 23 Oct 2023]. https://ec.europa.eu/eurostat/databrowser/view/hlth_ehis_ic1u__custom_8024987/default/table?lang=en&page=time:2019

[43] Cree VE. Worries and problems of young carers: issues for mental health. Child & Family Social Work. 2003;8(4):301–9. doi: 10.1046/j.1365-2206.2003.00292.x

[44] Robison OMEF, Inglis G, Egan J. The health, well-being and future opportunities of young carers: a population approach. Public Health. 2020;185:139–43. doi: 10.1016/j.puhe.2020.05.002 32622221

[45] Fleitas Alfonzo L, Singh A, Disney G, Ervin J, King T. Mental health of young informal carers: a systematic review. Soc Psychiatry Psychiatr Epidemiol. 2022;57(12):2345–58. doi: 10.1007/s00127-022-02333-8 35798995 PMC9263065

[46] de Zwart PL, Bakx P, van Doorslaer EKA. Will you still need me, will you still feed me when I’m 64? The health impact of caregiving to one’s spouse. Health Econ. 2017;26 (2):127–38. doi: 10.1002/hec.3542 28940916 PMC5639350

[47] Schulmann K, Reichert M, Leichsenring K. Social Support and Long-Term Care for Older People: The Potential for Social Innovation and Active Ageing. The Future of Ageing in Europe. Springer Nature Singapore. 2018. p. 255–86. doi: 10.1007/978-981-13-1417-9_9

[48] Robison JT, Shugrue NA, Fortinsky RH, Fabius CD, Baker K, Porter M, et al. A New Stage of the Caregiving Career: Informal Caregiving After Long-term Institutionalization. Gerontologist. 2021;61(8):1211–20. doi: 10.1093/geront/gnaa185 33170252

[49] Börsch-Supan A, Brandt M, Hunkler C, Kneip T, Korbmacher J, Malter F, et al. Data Resource Profile: the Survey of Health, Ageing and Retirement in Europe (SHARE). Int J Epidemiol. 2013;42(4):992–1001. doi: 10.1093/ije/dyt088 23778574 PMC3780997

