# Correction: Informal care in different European care systems: Effects of caregiving on mental health over time

**DOI:** 10.1371/journal.pone.0339047

**Published:** 2025-12-15

**Authors:** Leonie Börner, Ingo W. K. Kolodziej, Jürgen Wasem, Carina Abels

The second author, Ingo W. K. Kolodziej, is no longer available as corresponding author. The only corresponding author is Carina Abels. Carina Abels’s email address is: Carina.Abels@medman.uni-due.de.
