# Supplementary material for: Informal care in different European care systems: Effects of caregiving on mental health over time
Source: PLoS One. 2025 Oct 15;20(10):e0332498. doi: 10.1371/journal.pone.0332498 (PMC12527147; doi:10.1371/journal.pone.0332498)
Supplement: S1 Table — (PDF) [file pone.0332498.s001.pdf]

S1 Table. Description of used variables

Variable	Coding	Comment
Informal Care	0, 1 if individual performs almost daily support for at least three months (= informal care activities within the household) or if individual performs at least some hours of caregiving monthly (= informal care activities outside the household	Within the SHARE data, informal caregiving activities inside and outside the household include supportive activities, such as help with dressing, bathing, showering, etc., and refer to the last 12 months. Informal care activities within the household refer to daily or almost daily support services that were provided for at least three months. In contrast, the extent of informal care activities outside the household is surveyed separately and differentiated according to daily or almost daily, weekly, monthly, and less frequently.
**Mental Health**		
**EURO-D score**	**0-12**	
**4+ depressive symptoms**	**0, 1 if screen positive**	
**Parental information**		
Single parent	0, 1 if yes	
Mother in fair or poor health	0, 1 if yes	Within the SHARE data, the health status of the parents is classified by the respondent using the response options "very good", "good", "fair", "poor" and "very poor" in wave 1 and "excellent", "very good", "good", "fair" and "poor" in the following waves. According to Jürges/Avendano/Mackenbach (2008)[1], the combination of the categories "poor" and "very poor" as well as "excellent" and "very good" continues to provide a consistent measurement.
Father in fair or poor health	0, 1 if yes	
Mother alive	0, 1 if yes	
Father alive	0, 1 if yes	
**Health**		
Number of chronic diseases	count	
ADL limitations	0-6	
IADL limitations	0-6	
Sociodemographics		
Age	In years	
Married	0, 1 if yes	
Household size	Headcount	
Partner in household	0, 1 if yes	
Employed	0, 1 if yes	
Financial difficulties	0, 1 if yes	

[1] Jürges, H./Avendano, M./Mackenbach, J. P. (2008): Are different measures of self-rated health comparable? An assessment in five European countries. In: European Journal of Epidemiology, Jahrgang 23, Heft 12, S. 773–781
